# An Unusual Case of Myelodysplastic Syndrome With Intrahepatic Extramedullary Hematopoiesis Leading to Liver Failure

**DOI:** 10.7759/cureus.22733

**Published:** 2022-03-01

**Authors:** Logan Ternes, Faye Giangiacomo, Imad Nassif

**Affiliations:** 1 Internal Medicine, Medical Center of Trinity, Trinity, USA; 2 Pathology, Ascension Via Christi St. Francis Hospital, Wichita, USA; 3 Gastroenterology and Hepatology, Wichita Endoscopy Center, Wichita, USA

**Keywords:** extramedullary hematopoieisis, myelodysplastic syndrome, pancytopenia, bilirubin, cholestasis

## Abstract

We present a rare case of a 61-year-old male who arrived at the hospital with fatigue and was found to have pancytopenia. Following further workup, he was diagnosed with myelodysplastic syndrome. His initial bilirubin of 3.1 mg/dL rose to 38.0 mg/dL. After a liver biopsy was obtained, intrahepatic extramedullary hematopoiesis (EMH) was diagnosed and showed the liver to be infiltrated by EMH without malignancy. Within 16 days of his stay, he developed liver failure and passed away. Although intrahepatic EMH is uncommon, EMH resulting in infiltration, liver failure, and then death is exceedingly rare.

## Introduction

Myelodysplastic syndrome (MDS) is a group of disorders within the bone marrow that causes dysplasia and ineffective hematopoiesis [1]. It is estimated that the incidence of MDS ranges between 5.3 and 13.1 per 100,000, and MDS prevalence in the United States is estimated to be between 60,000 and 170,000 [2]. Extramedullary hematopoiesis (EMH) is the formation of cellular blood components outside of the bone marrow, normally occurring in the liver and spleen in a fetus [3]. This often occurs in patients with chronic anemia. There have been around 30 reports of presacral EMH, as well as one such case of presacral EMH with MDS [4]. Though these reports exist, it is additionally very rare for MDS to occur with intrahepatic EMH. To our knowledge, only one such case that had a mass-forming lesion in the context of iron overload in the liver has been published [5]. We present a case of a 61-year-old Caucasian male with a non-mass-forming lesion in the setting of MDS.

## Case presentation

A 61-year-old Caucasian male presented to the hospital with weakness, fatigue, and exertional shortness of breath for three weeks. Upon admission, he was found to have pancytopenia with elevated total bilirubin. Computerized tomography (CT) scan of the abdomen and pelvis revealed fluid around the liver and spleen with splenomegaly. Bone marrow biopsy showed marked pancytopenia with 7.5% blasts, trilineage hematopoiesis with adequate iron storage, and no increased ring sideroblasts seen. MDS with excess blasts was diagnosed with no evidence of lymphoma. During his stay, his bilirubin continued to rise from 3.1 up to 38.0 mg/dL. Due to his thrombocytopenia, a liver biopsy was not able to be performed until an adequate platelet level was achieved. Magnetic resonance imaging (MRI) of the abdomen and magnetic resonance cholangiopancreatography (MRCP) showed cholelithiasis without intrahepatic or extrahepatic biliary ductal dilatation, no filling defects in the common bile duct, hepatic steatosis, and morphologic changes of cirrhosis with sequela of portal hypertension. Extensive work-up for causes of liver pathologies including nonalcoholic fatty liver disease, autoimmune hepatitis, acute myeloid leukemia, sarcoidosis, and intrahepatic hematologic neoplasm were performed.

**Figure 1:**
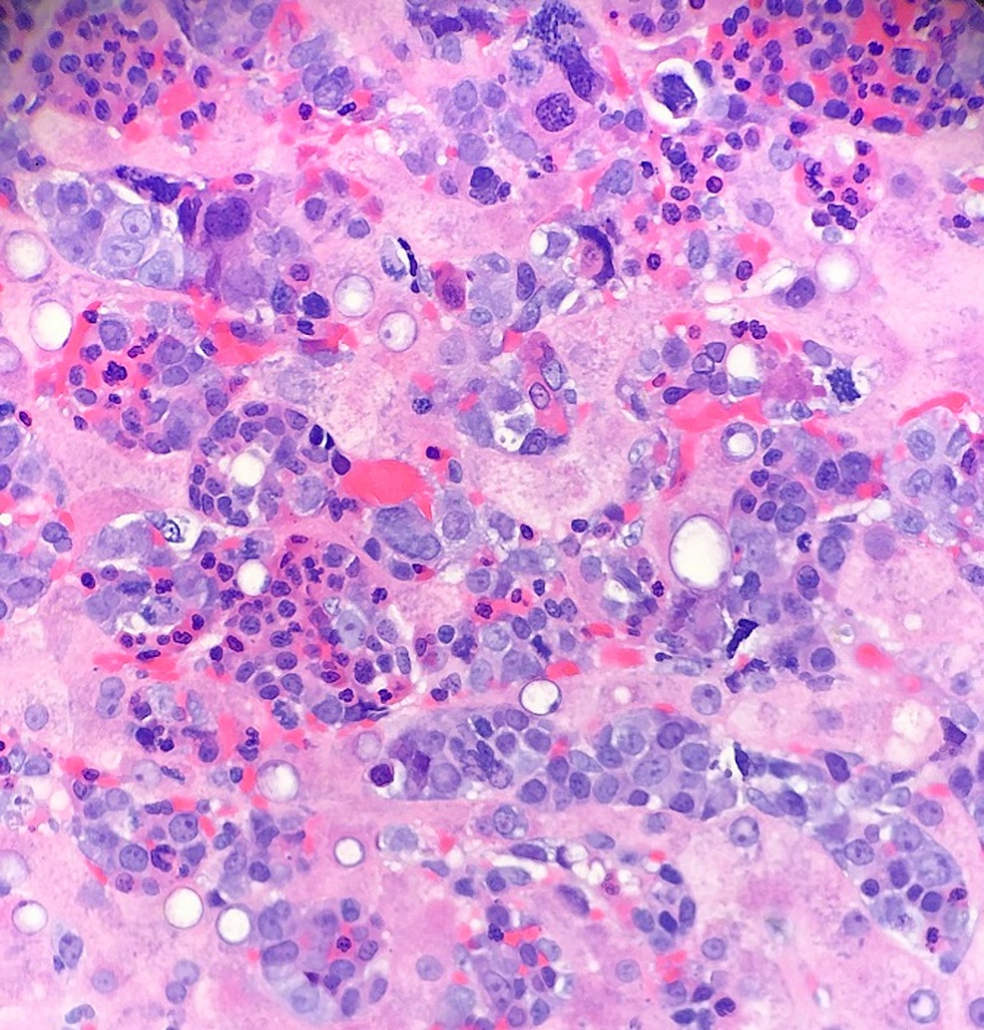
40X magnification picture The above image shows the liver tissue diffusely involved by extramedullary hematopoiesis (EMH) (H&E stain).

**Figure 2:**
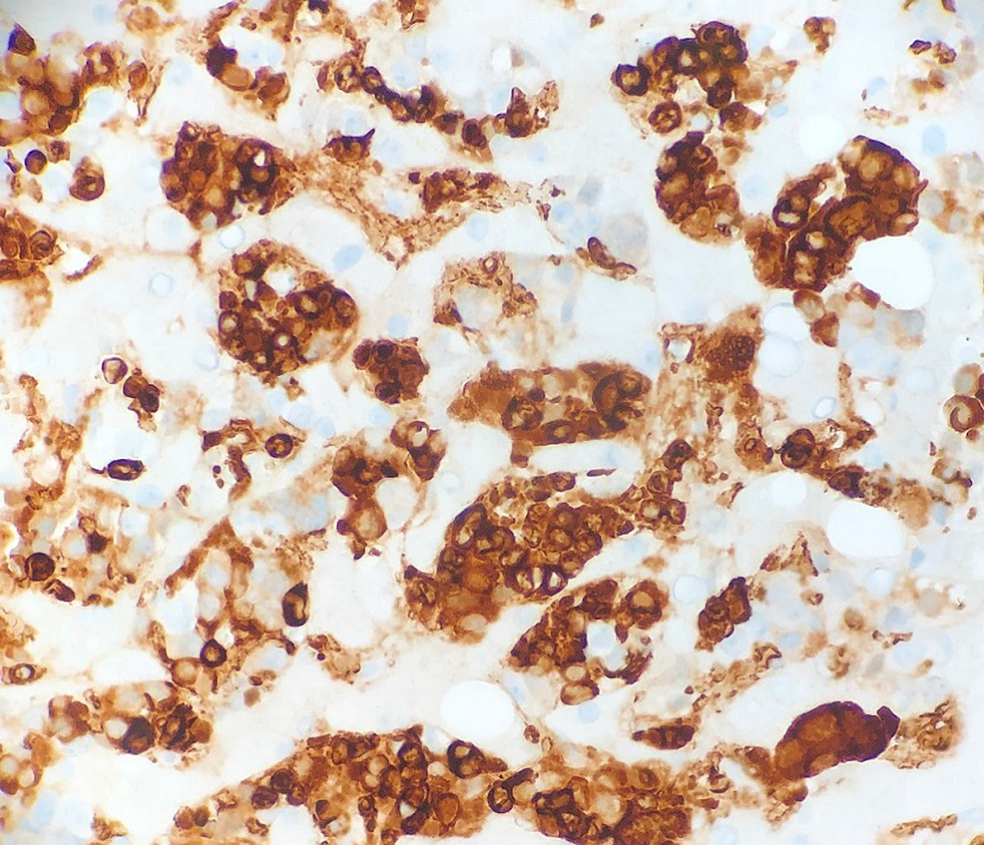
Hemoglobin immunohistochemical stain picture The majority of EMH cells are erythroid precursors (positive staining with hemoglobin immunohistochemical stain).

Peripheral blood smear showed 6% blasts. Pertinent lab ranges were as seen in Tables 1-2. Flow leukemia panel showed peripheral blood 12% blasts consistent with a clonal myeloid neoplasm. When stable, a liver biopsy was obtained from the patient and can be seen in Figures 1-2. The surgical pathology report showed the liver involved by marked erythroid predominant extramedullary hematopoiesis, hepatocanalicular cholestasis, marked hemosiderosis in hepatocytes (parenchymal pattern), periportal and bridging fibrosis, no lymphoblasts or myeloblasts, and no infiltrating carcinoma. During his stay in the hospital, his liver failure continued to worsen. He was unable to receive a liver transplant. Unfortunately, he passed away on the 16th day at the hospital.

**Table 1:** Biochemistry WBC: white blood cells, HgB: hemoglobin, ALT: alanine aminotransferase, AST: aspartate aminotransferase, Alk phos: alkaline phosphatase, LDH: lactate dehydrogenase,  INR: international normalized ratio, TIBC: total iron-binding capacity

Laboratory Test	Lab Value	Reference Range
HgB (g/dL)	6.3-8.0	14-18
WBC (K/uL)	1.4-3.9	4.5-11
Platelets (K/uL)	6-45	150-450
Direct Coombs (positive/negative)	negative	negative
Iron (ug/dL)	65	50-150
TIBC (ug/dL)	231	250-310
Iron saturation (%)	28	20-50
Transferrin (mg/dL)	155	200-400
LDH (U/L)	2409-3727	80-225
Haptoglobin (mg/dL)	8-11	83-267
INR	1.3-2.3	<1.1
D-dimer (ng/mL FEU)	1,879	<250
Total bilirubin (mg/dL)	3.1-38.0	0.3-1.0
ALT (U/L)	127-390	10-40
AST (U/L)	62-160	10-40
Alk phos (U/L)	78-175	30-120
Albumin (g/dL)	2.1-2.6	3.5-5.5
Total protein (g/dL)	4.0-5.3	5.5-9.0

**Table 2:** Immunological Tests ACE: angiotensin-converting enzyme, Ig: immunoglobulin, EBV: Epstein-Barr Virus, CMV: cytomegalovirus, Ag: antigen

Laboratory Test	Lab Value	Reference Range
Alpha fetoprotein (ng/mL)	1.9	<10 ng/mL
Antinuclear antibodies (positive/negative)	Negative	Negative
Smooth muscle antibodies (positive/negative)	Negative	Negative
Double-stranded DNA antibodies (positive/negative)	Negative	Negative
Endomysial IgA (positive/negative)	Negative	Negative
Mitochondrial antibodies (positive/negative)	Negative	Negative
ACE (u/L)	Unobtainable due to interfering substance present	8-53
EBV capsid IgG (positive/negative)	Positive	Negative
CMV DNA (IU/mL)	Not Detectable	Not Detectable
Hepatitis A IgM (positive/negative)	Negative	Negative
Hepatitis BsAg (positive/negative)	Negative	Negative
Hepatitis B core IgM (positive/negative)	Negative	Negative
Hepatitis C antibody (positive/negative)	Negative	Negative

## Discussion

The combination of MDS with intrahepatic extramedullary hematopoiesis is a rare occurrence. EMH appears in many disorders, including myeloproliferative conditions. A diagnosis of MDS must be differentiated from myelofibrosis in these situations, which can be done by examining the bone marrow. Myelofibrosis occurs when fibrous scar tissue replaces the bone marrow and is thought to be due to genetic mutations [6]. It can present with anemia, leukocytosis, thrombocytopenia or thrombocytosis, hepatosplenomegaly, and EMH [6]. MDS is differentiated by having cytopenias with dysplastic morphology. EMH happens when the function of the bone marrow is insufficient and may occur in locations such as the liver [7]. Although this patient did have intrahepatic EMH, it is not often seen in combination with myelodysplasia [4].

Belay et al. described a similar incident of MDS with intrahepatic EMH [5]. Their case is different from ours as they reported a patient with a noticeable mass within their liver. The importance of their case report was to describe the usefulness of T2*-weighted gradient echo imaging in the diagnosis of mass-forming hepatic extramedullary hematopoiesis arising in the setting of diffuse iron overload [5]. The importance of our case shows that EMH within the liver may not coincide with a mass but is able to induce cholestasis and rapid liver failure. EMH was only diagnosed in our patient after a biopsy was taken, which can be seen in Figures 1-2.

Cholestasis can occur due to many conditions, including pregnancy, bile duct tumors or stones, primary biliary cholangitis, primary sclerosing cholangitis, sarcoidosis, drugs, and alcoholic liver disease [8]. Cholestasis in the setting of EMH and rapid worsening of this patient’s liver failure in the absence of a hepatic infiltrative malignant process is unusual after reviewing the literature.

## Conclusions

This case shows that it is possible to have cholestasis with EMH and rapid demise from liver failure. We believe this to be a rare incidence of a non-mass forming diagnosis of intrahepatic EMH with MDS. In future cases, when unknown or when considering a diagnosis such as MDS with EMH, it is important to stabilize the patient’s platelet count to be able to obtain a tissue sample so as not to delay treatment or other options. Thus, as an abnormal presentation of MDS, it is necessary to keep the consideration of intrahepatic EMH causing cholestasis in the differential diagnosis.
